# Exercise as an Adjuvant to Cartilage Regeneration Therapy

**DOI:** 10.3390/ijms21249471

**Published:** 2020-12-12

**Authors:** John Kelly Smith

**Affiliations:** Departments of Academic Affairs and Biomedical Sciences, James H. Quillen College of Medicine, East Tennessee State University, P.O. Box 70300, Johnson City, TN 37614, USA; smithj@etsu.edu; Tel.: +1-423-794-7425

**Keywords:** exercise, osteoarthritis, osteoporosis, mesenchymal stem cells, hematopoietic stem cells, stem cell transplantation, chondroblasts, chondrocytes, cytokines

## Abstract

This article provides a brief review of the pathophysiology of osteoarthritis and the ontogeny of chondrocytes and details how physical exercise improves the health of osteoarthritic joints and enhances the potential of autologous chondrocyte implants, matrix-induced autologous chondrocyte implants, and mesenchymal stem cell implants for the successful treatment of damaged articular cartilage and subchondral bone. In response to exercise, articular chondrocytes increase their production of glycosaminoglycans, bone morphogenic proteins, and anti-inflammatory cytokines and decrease their production of proinflammatory cytokines and matrix-degrading metalloproteinases. These changes are associated with improvements in cartilage organization and reductions in cartilage degeneration. Studies in humans indicate that exercise enhances joint recruitment of bone marrow-derived mesenchymal stem cells and upregulates their expression of osteogenic and chondrogenic genes, osteogenic microRNAs, and osteogenic growth factors. Rodent experiments demonstrate that exercise enhances the osteogenic potential of bone marrow-derived mesenchymal stem cells while diminishing their adipogenic potential, and that exercise done after stem cell implantation may benefit stem cell transplant viability. Physical exercise also exerts a beneficial effect on the skeletal system by decreasing immune cell production of osteoclastogenic cytokines interleukin-1β, tumor necrosis factor-α, and interferon-γ, while increasing their production of antiosteoclastogenic cytokines interleukin-10 and transforming growth factor-β. In conclusion, physical exercise done both by bone marrow-derived mesenchymal stem cell donors and recipients and by autologous chondrocyte donor recipients may improve the outcome of osteochondral regeneration therapy and improve skeletal health by downregulating osteoclastogenic cytokine production and upregulating antiosteoclastogenic cytokine production by circulating immune cells.

## 1. Introduction

In the global burden of disease 2010 study, osteoarthritis accounted for 17,135 years of life lived with disability (YLDs), an increase of 64% when compared to YLDs of 1990. Overall, musculoskeletal disorders (which included inflammatory causes of arthritis) accounted for 6.8% of total YLDs [1], with osteoarthritis ranked as the 11th leading cause of disability worldwide [2]. The global prevalence of osteoarthritis is expected to increase as the average age and weight of the world’s population increases.

Physical exercise has long been recognized as an essential factor in the maintenance of skeletal health, particularly during adolescence, when ~50% of bone mass accretion occurs [3]. The 2019 American College of Rheumatology/Arthritis Foundation guidelines for the management of osteoarthritis of the hip and knee emphasized the importance of regularly performed physical exercise [4]. Both traditional (resistance, aerobic, and flexibility) and non-traditional (tai chi, yoga, and aquatic) exercises have been shown to be effective in the management of knee and hip osteoarthritis [5]. In their systematic review of 44 clinical trials involving patients with knee osteoarthritis, Fransen and associates found that land-based therapeutic exercises reduced pain and improved physical function and the quality of life for at least 2–6 months after the cessation of formal treatment [6]. In this regard, the World Health Organization recommends that adult men and women should accumulate at least 150 min of moderate intensity physical exercise per week and young people aged 5–17 years should accumulate at least 60 min of physical exercise of moderate to vigorous intensity daily [7].

There is increasing interest in treating articular cartilage and subchondral bone defects and osteoarthritis with autologous chondrocyte implants (ACIs), matrix autologous chondrocyte implants (MACIs), and bone marrow-derived mesenchymal cell implants or injections [8,9,10,11,12,13,14]. ACI and MACI procedures have been shown to produce durable long-term outcomes in the treatment of partial and full-thickness articular cartilage defects in tibiofemoral joints [15,16,17,18,19]. In addition, mesenchymal stem cells mobilized to joints from the peripheral blood or placed on implantation matrices have the potential to repair cartilage by differentiating into chondrocytes [20].

In this article, I review the pathophysiology of osteoarthritis and detail how physical exercise improves the health of articular cartilage and chondrocytes in osteoarthritis and enhances the potential of mesenchymal stem cells and chondrocytes for successful implantation therapy. I discuss how exercise protects the skeletal system by upregulating the production of antiosteoclastogenic cytokines and downregulating the production of osteoclastogenic cytokines by chondrocytes and peripheral blood mononuclear cells. A summary of scaffolds currently approved or undergoing clinical trials for MACI and exercise protocols that can be used to rehabilitate osteoarthritis patients and recipients of chondrocyte and mesenchymal stem cell implants is provided. I also provide an aerobic exercise protocol that can be used to condition tissue donors.

## 2. Materials and Methods

This narrative review is on exercise as an adjuvant to cartilage regeneration therapy. The research strategy included the following: 1. defining key topics; 2. identifying key words or synonyms that represent each of the key topics; 3. an online PubMed search of key topics and key words; and 4. a refinement of the search based on initial findings. The search included all reviews, meta-analyses, and clinical trials on key topics published between the years 2000 and 2020. Restricted were review articles with identical samples and identical outcomes, identical samples with different outcomes, increased samples and identical outcomes, and decreased samples with identical outcomes. Key topics included osteoarthritis, exercise and osteoarthritis, stem cells, stem cell implants, exercise and stem cells, mesenchymal stem cells, mesenchymal stem cell implants, exercise and mesenchymal stem cells, hematopoietic stem cells, hematopoietic stem cell implants, exercise and hematopoietic stem cells, chondrocytes, chondrocyte implants, autologous chondrocyte implantation, matrix-induced autologous chondrocyte implantation, exercise and chondrocytes, osteoclastogenic cytokines, exercise and osteoclastogenic cytokines, implantation scaffolds, antiosteoclastogenic cytokines and exercise, and antiosteoclastogenic cytokines. Keywords included exercise, osteoarthritis, osteoporosis, stem cells, mesenchymal stem cells, hematopoietic stem cells, chondrocytes, osteoclastogenic cytokines, and antiosteoclastogenic cytokines.

## 3. Definition of Exercise

In this article, the author has used the following definition of exercise: a physical activity that involves repetitive voluntary contractions of limb, back, or abdominal muscles that are of sufficient force to maintain or improve physical conditioning.

## 4. Pathophysiology of Osteoarthritis

Osteoarthritis (OA) is a complex polygenetic disease involving structural and functional alterations of the entire joint, including the articular cartilage, menisci, subchondral bone, capsule, synovium, ligaments, and periarticular muscles. The knee is the most common joint to be involved, followed by the hand and hip. OA is especially prevalent in the elderly, particularly elderly women, in overweight individuals, and in persons with traumatic joint injuries, including those that are work related (e.g., lifting of heavy loads, constant knee bending). The contribution of genetics in OA is estimated to be between 40–80%, with late onset OA being associated with many common DNA variants [21].

OA is an ongoing process involving mechanical, inflammatory, and metabolic derangements of the articular cartilage, subchondral bone, and synovium. This is reflected in the finding of elevated blood levels of C-reactive protein (CRP) and picogram amounts of the proinflammatory cytokines tumor necrosis factor (TNF)-α, interleukin (IL)-6, and IL-1β in OA that are present early in the disease and proportionate to the patients’ symptoms and, in the case of TNF-α, in keeping with radiographic findings of OA [22].

Articular cartilage primarily comprises tissue fluid, which accounts for 65–85% of its mass, and type II collagen and proteoglycans, which account for 15–22% and 4–7% of its mass, respectively. Proteoglycans include the small leucine-rich decorin, lumican, biglycan, fibromodulin, lumican, and epiphycan, and the heparin sulfate proteoglycan perlecan. Other collagens (types V, VI, IX, X, XI, XII, IV), cell adhesins, growth factors, and cytokines are also present. The predominant cell in articular cartilage is the chondrocyte, which is responsible for maintaining articular homeostasis by replacing degraded matrix with newly synthesized components [23]. In articular cartilage, synovium, and synovial fluid, mesenchymal stem cells are also present and may serve as a valuable source of stem cell transplantation in the treatment of OA [24].

Osteoarthritis is characterized by early alterations in the organization and molecular composition of the articular cartilage extracellular matrix. This change is met with a compensatory proliferative response of chondrocytes and an increase in chondrocyte matrix synthesis. With time, hypertrophied chondrocytes become senescent, a phenotype associated with the secretion of proinflammatory cytokines and matrix-degrading proteases and a reduction in the secretion of anti-inflammatory cytokines [25,26,27]; this, in turn, stimulates proliferative and proinflammatory responses in adjacent synovium and periarticular bone, causing synovial hypertrophy and contributing to the development of osteophytes. Subchondral bone turnover and angiogenesis is increased, with consequent vascular invasion of the cartilage [21]. Senescent chondrocytes eventually undergo apoptosis, terminating articular cartilage synthesis [25]. Other metabolic derangements involving transforming growth factor (TGF)-β, fibroblast growth factor (FGF)-2, FGF-18, growth differentiation factor (GDF)-5, and hypoxia-induced factor (HIF)-2a may also contribute to the pathogenesis of OA [23].

Studies on menisci, which contain multiple subpopulations of cells responsible for tissue repair and maintenance (“fibrochondrocytes”) have shown that their production of IL-1 is elevated in OA (109–288 pg/mL). IL-1 is a potent proinflammatory and osteoclastogenic cytokine whose effects on menisci and articular cartilage are catabolic [28].

**Figure 1:**
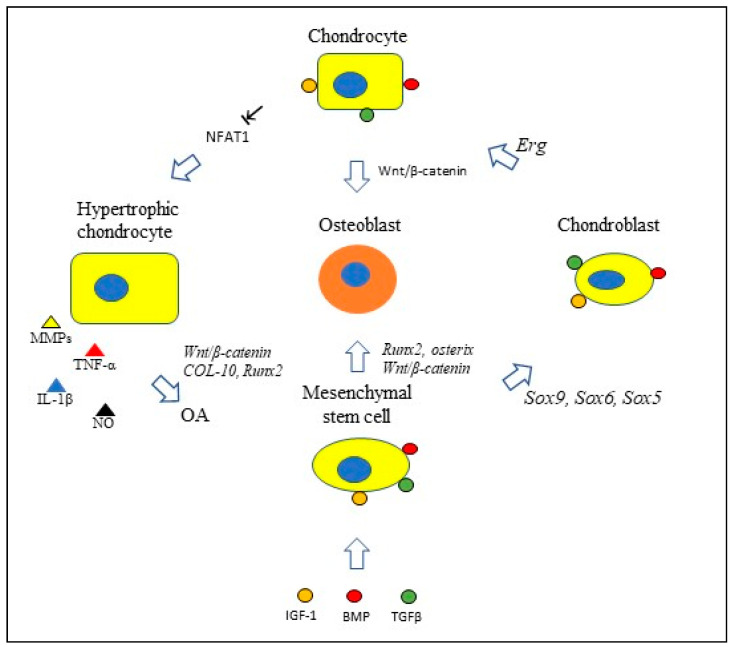
With the aid of growth factors IGF-1, TGF-β, and bone morphogenic proteins (BMPs), bone marrow mesenchymal stem cells expressing *SRY-related HMG-Box9 (Sox9)* and/or *Sox6 or Sox5* differentiate into chondroblasts. *Ets-related gene* (*Erg*) transcriptional activation prompts chondroblasts to differentiate into mature chondrocytes expressing the transcription factor NFAT1, which plays a critical role in maintaining chondrocyte homeostasis. In adult mice, deletion of chondrocyte NFAT1 results in a loss of type II collagen and aggrecan and an over-expression of cartilage-degrading matrix metalloproteinases (MMPs), proinflammatory cytokines, and nitric oxide (NO); this is accompanied by chondrocyte proliferation and hypertrophy, destruction of articular surfaces, osteophyte formation, and exposure of subchondral bone—findings characteristic of osteoarthritis (OA).

Studies in mice indicate that nuclear factor of activated T cells 1 (NFAT1), a member of the NFAT transcription factors, plays a critical role in maintaining the anabolic functions of adult articular chondrocytes by regulating their expression of matrix-degrading proteinases and proinflammatory cytokines. Deletion of NFAT1 in adult mice results in a loss of type II collagen and aggrecan and an over-expression of matrix-degrading proteases and proinflammatory cytokines; this is followed by chondrocyte proliferation and hypertrophy, destruction of articular surfaces, osteophyte formation, and exposure of subchondral bone—findings characteristic of OA [29]. Rodova and associates determined that NFAT1 expression in articular cartilage is regulated epigenetically by histone methylation [30]. The anabolic activity of chondrocytes is maintained by their secretion of growth factors TGF-β, insulin-like growth factor (IGF)-1, FGF-2, FGF-18, GDF-5, and bone morphogenic proteins (BMPs) (Figure 1).

Wnt/β-catenin signaling in chondrocytes can prompt their differentiation into osteoblasts and the activation of runt-related transcription factor-2 (*Runx2)* and osterix (Osx) in mesenchymal stem cells prompts their differentiation into osteoblasts. Other growth factors involved in chondrocyte homeostasis are FGF-2, FGF-18, and GDF-5.

## 5. Exercise and Osteoarthritis

Although regularly performed moderate-intensity exercise is recognized as the mainstay treatment of OA [4,5], there are a limited number of studies sampling constituents of the OA joint before and after supervised exercise training of men and women. One of these was published by Roos and Dahlberg and involved 45 subjects who had undergone medial meniscus resection 3–5 years prior to the study and were at risk of developing OA. Subjects underwent supervised exercise training three times weekly for 4 months or were assigned to a noninterventional group. All subjects had the glycosaminoglycan content of their knee cartilage assessed by delayed gadolinium-enhanced magnetic resonance imaging. Exercise increased cartilage levels of glycosaminoglycan in proportion to the level of physical activity [31].

In a similar study, Munukka and associates assessed the effects of 12 months of leisure time physical activity on the glycosaminoglycan content of femoral cartilages in 76 post-menopausal women with knee OA using delayed gadolinium-enhanced magnetic resonance imaging. They also found that exercise increased the amount of cartilage glycosaminoglycan [32].

Iijima and associates studied the effects of 2–4 weeks of treadmill walking in 24 male Wistar rats with induced damage to their knee joints using micro-computed tomography, histology, and immunohistochemistry analysis. They found that exercise prevented the progression of post-traumatic bone and cartilage lesions and increased BMP-2 and BMP-6 expression in the joint superficial zone chondrocytes [33].

Assis and associates studied the effects of aerobic exercise training on an experimental model of knee osteoarthritis in 50 male Wistar rats. Twenty of the rats were trained on treadmills 3 days/week at 16 m/min for 50 min/day for 8 weeks. The exercising and control rats were sacrificed, and their knee joints assessed by histologic, morphometric, and immunohistochemical analysis. Compared to the controls, exercising animals had a better pattern of cartilage organization and less cartilage degeneration. Exercising animals also had lower chondrocyte nuclear or nucleolar expression of IL-1β, caspase-3, and MMP-13, confirming the ability of aerobic exercise to downregulate proinflammatory and proteolytic pathways in this model of OA [34].

## 6. Exercise and Mesenchymal Stem Cells

The author is using the International Society for Cell and Gene Therapy committee’s recommendation that the acronym “MSC” be used for both mesenchymal stem cells and mesenchymal stromal cells and that the MSC acronym be preceded by “BM” for bone marrow origin and “AD” for adipose tissue origin [35].

### 6.1. Exercise Studies in Rodents

**Figure 2:**
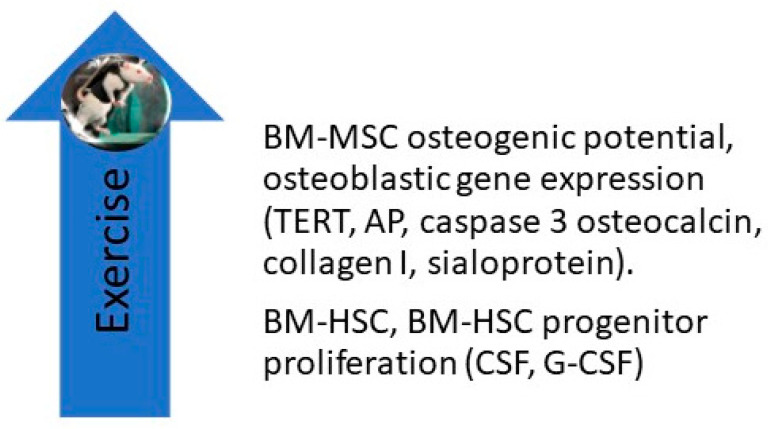
Studies in rodents have shown that treadmill exercise upregulates the osteogenic potential of BM-MSCs, including their expression of osteogenic genes. Exercise also increases the proliferative capacity of BM-HSC and BM-HSC progenitors by upregulating G-CSF and CSF levels in their secretome. AP, alkaline phosphatase; BM-HSC, bone marrow-derived hematopoietic stem cell; CSF, colony-stimulating factor; G-CSF, granulocyte colony stimulating factor; TERT, telomerase reverse transcriptase.

Liu et al. reported the effects of 8 weeks of treadmill exercise (60 min per day at 19.3 m/min, 5-degree incline) on the proliferative, differential, and apoptotic abilities of cultured femoral bone marrow-derived mesenchymal stem cells (BM-MSCs). They found that exercise enhanced their osteogenic potential and decreased their adipogenic potential (Figure 2) and posited that “BMSC derived from exercised rats on early passage may be a good cell source for bone tissue engineering” [36]. Emmons et al. report that 15 and 60 min of treadmill exercise done by C57 BI/6 mice increased the proliferative capacity of their bone marrow hematopoietic stem cells (BM-HSCs) and multipotential HSC progenitors by 40–61%. They attribute these findings to a change in the BM-HSC secretome, which included an upregulation of granulocyte colony-stimulating factor (G-CSF) and stem cell factor (SCF) [37]. Bourzac et al. reviewed literature reports on the effects of physical exercise on MSC proliferation, differentiation, and homing and found that the effects of exercise varied depending on the exercise protocol and the tissue from which MSCs were obtained; they concluded that “the combination of physical exercise and MSC engraftment improves neural, cartilage, and muscular tissue recovery, but it is not clear whether the effects of MSCs and exercise are additive or synergistic” [38]. Ocarino et al. studied the effects of exercise on BM-MSCs in osteopenic female Wistar rats with and without nitric oxidase inhibition. BM-MSCs were isolated from their femurs and cultured in osteogenic medium for 7, 14, and 21 days, phenotyped, and analyzed for alkaline phosphatase, collagen synthesis, and the formation of mineralized nodules. They found that exercise increased BM-MSC osteogenesis and that inhibition of nitric oxide diminished their osteogenic response. They concluded that “nitric oxide mediates the beneficial effects of physical activity upon MSCs osteogenic differentiation” [39]. Hell and associates measured the effects of treadmill exercise on the osteogenic potential of BM-MSCs in young and adult female Wistar rats by measuring cell viability, percentage of cells per field, mineralized nodular number, and gene expression for telomerase reverse transcriptase (TERT), alkaline phosphatase (AP), caspase 3, osteocalcin, collagen I, and sialoprotein. They found that exercise increased the differentiation of BM-MSCs in both study groups, but the effect was greater in young animals than in adults [40]. Using mice, Wallace and associates measured the effects of 5 days of treadmill exercise (30 min/day) on BM-MSCs and found that exercise increased their osteogenic potential [41]. Yamaguchi measured the effects of exercise on the ability of BM-MSCs obtained from male Wistar rats to repair experimentally induced femoral groove osteochondral defects in female Wistar rats. Two weeks after BM-MSCs were injected into the defective joints, rats were either sedentary or subjected to 2, 4, or 8 weeks of treadmill exercises performed 5 days/week at 12 m/min for 30 min; the animals were then sacrificed, and their joints subjected to immuno-histochemical staining. Compared to the sedentary group, they found that exercise enhanced cartilage repair and concluded that their study “highlights the importance of exercise following cell transplantation therapy” [42].

### 6.2. Exercise Studies in Humans

There are a limited number of studies on the effect of exercise on human BM-MSCs. Schmidt and associates studied the effects of short-term high-intensity exercise on the ability of post-exercise sera to influence the proliferation, migration, and apoptosis activity of cultured BM-MSCs. They found that post-exercise sera enhanced the migratory capacity of BM-MSCs, a finding they attributed to the generation of IL-6 by contracting skeletal muscles. They posited that “there is a direct relationship between exercise, IL-6 release and stem cell recruitment” [43]. Carbonare et al. studied the effects of running one-half of a marathon on the differentiation potential of mesenchymal circulating progenitor cells (M-CPCs) and on the effects of sera on a human bone marrow-derived mesenchymal stem cell (hBM-MSC) line in 22 athletes. They found that exercise upregulated the expression of osteogenic genes *Runx2, muscle segment homeobox gene 1 (MSx1), secreted phosphoprotein 1 (SPP-1)*, and chondrogenic genes *SRY-Box 9 (SOX9)* and *collagen type II alpha-1 gene (COL2A1),* and apoptosis-related genes *autophagy-related gene 3* *(ATG3)* and *Unc-like kinase gene (Ulk1)* in M-CPCs. BMP2 and BMP6 were also upregulated. The authors concluded that exercise upregulated the differentiation and apoptosis of BM-MSCs [44]. In a study involving 20 amateur runners, Valenti et al. assessed the effects of running one-half of a marathon on the expression of microRNAs (miRNAs) in human BM-MSCs incubated with pre- and post-exercise sera. They found that exercise upregulated the expression of miRNAs promoting osteoblast differentiation, including miR-21-5p, miR-129-5p, miR-378-5p, and miR-188-5p, while downregulating the expression of a miRNA that promotes adipocyte differentiation (miRNA-188-5p). They also found that exercise upregulated the expression of the osteogenic gene *Runx2* [45]. Niemiro et al. studied the kinetics of progenitor cell mobilization during 60 min of treadmill exercises (70% Vo_2peak_) performed by seven men. They found that exercise increased circulating levels of cysteine x cysteine (CXC) chemokine ligand (CXCL)-12 and SCF in hematopoietic stem cells but not in BM-MSCs. They concluded that exercise may serve as a valuable adjunct in the context of HSC transplants [46].

**Figure 3:**
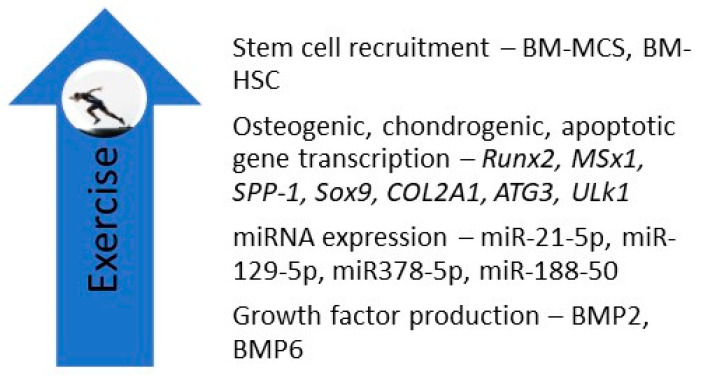
Studies in humans indicate that exercise increases the recruitment of BM-MSCs and BM-HSCs and upregulates BM-MSC expression of osteogenic, chondrogenic, and apoptotic genes, osteogenic microRNAs, and osteogenic growth factors. *ATG3, autophagy-related gene 3; COL2A1, collagen type II alpha-1 gene; MSx1, muscle segment homeobox gene 1; SPP-1, secreted phosphoprotein 1; SOX-9; Ulk1, Ul kinase gene 1;* miR, microRNA.

In summary, experiments in humans, while limited in number, have shown that exercise upregulates BM-MSC and BM-HSC recruitment, enhances BM-MSC osteogenic, chondrogenic, and apoptotic gene expression, and upregulates BM-MSC expression of osteogenic miRNAs and the secretion of growth factors (Figure 3).

## 7. Mechanical Strain and Mesenchymal Stem Cells

### 7.1. In Rodents

**Figure 4:**
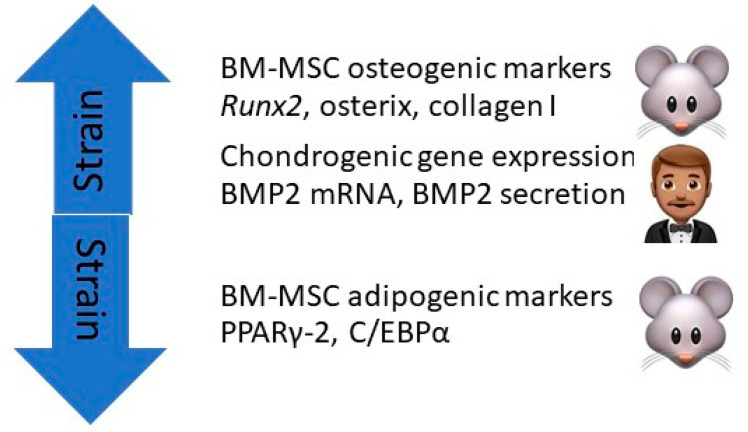
Mechanical strain exerted on rodent BM-MSC cultures by a FLEXcell-500 device increased their expression of osteogenic markers *Runx2,* osterix, and type I collagen and decreased their expression of adipogenic markers PPARγ-2 and C/EBPα. Mechanical strain exerted on human BM-MSCs increased their chondrogenic gene expression, and BMP2 mRNA and BMP2 secretion.

Using cultures of BM-MSCs flushed from the femurs and tibia of Sprague–Dawley rats, Runguang et al. found that mechanical strain exerted on the cultures by a FLEXcell-500 device increased BM-MSC expression of osteogenic markers *Runx2,* osterix (Osx), and type I collagen, and decreased their expression of adipogenic markers peroxisome proliferator-activated receptor-γ (PPARγ-2) and CCAAT enhancer-binding protein α (C/EBPα). *Runx2* is the main regulatory gene controlling skeletal development and morphogenesis in vertebrates, Osx is a transcription factor for osteoblasts, PPARγ-2 is a transcription factor that regulates differentiation of MSCs into adipocytes, and C/EBPα induces spleen focus-forming virus proviral integrin 1 (PU.1) and interacts with activator protein-1 (AP-1) and nuclear factor kappa-B (NF_K_B) to regulate myeloid development. The authors concluded that mechanical strain promotes BM-MSC differentiation into osteoblasts while impeding their differentiation into adipocytes [47] (Figure 4).

### 7.2. In Humans

Zhang et al. found that dynamic compression of the type that occurs with exercise increased the expression of chondrogenic genes in cultures of human BM-MSCs [48]. Sumanasinghe and associates seeded human BM-MSCs in 3D type I collagen matrices and subjected them to 0%, 10%, or 12% uniaxial cyclic tensile strain at 1 Hz for 4 h/day for 7 or 14 days. They found that BMP2 mRNA expression and BMP2 production were upregulated in the strain samples as compared to controls, indicating that mechanical strain of the type associated with exercise can induce osteogenic differentiation of human BM-MSCs [49] (Figure 4).

## 8. Exercise and Osteoclastogenic and Antiosteoclastogenic Cytokines

**Figure 5:**
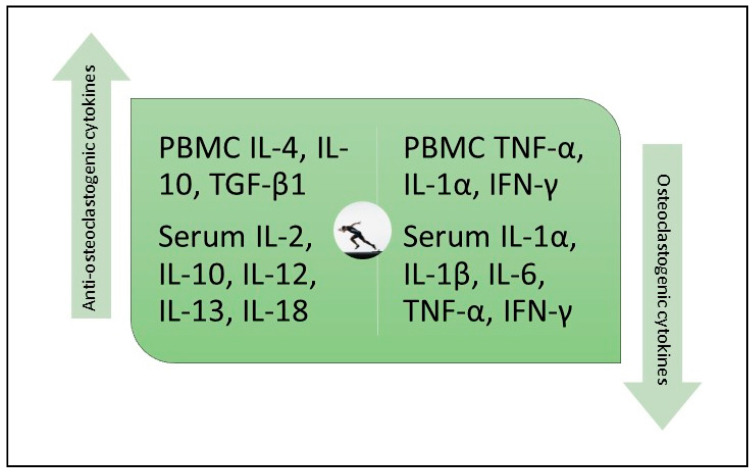
Exercise increases PBMC secretion and serum levels of antiosteoclastogenic cytokines, while reducing PBMC secretion and serum levels of osteoclastogenic cytokines. PBMC, peripheral blood mononuclear cell; IL, interleukin; TGF-β, transforming growth factor-β; TNF-α, tumor necrosis factor-α; IFN-γ, interferon-γ. IFN-γ is a pleotropic cytokine with both antiosteoclastogenic and osteoclastogenic activities which are context dependent.

In a before and after trial involving 43 healthy adults, Smith and associates measured the effect of six months of combined aerobic, resistance, and flexibility exercises on the production of osteoclastogenic cytokines (IL-1α, TNF-α), antiosteoclastogenic cytokines (TGF-β, IL-4, IL-10), and cytokines with variable effects on osteoclastogenesis (interferon (IFN)-γ, IL-6) by cultured mitogen-stimulated peripheral blood mononuclear cells (PBMCs). Serum markers of bone formation (osteocalcin) and bone resorption (C-terminal telopeptides of type I collagen) were also measured. Exercises done for 2.5 h a week on average attenuated the production of osteoclastogenic cytokines and enhanced the production of antiosteoclastogenic cytokines (Figure 5). These changes were accompanied by a 16% reduction in collagen degradation products and a 9.8% increase in osteocalcin levels. They concluded that “Long-term moderate intensity exercise exerts a favorable effect on bone resorption by changing the balance between blood mononuclear cells producing osteoclastogenic cytokines and those producing antiosteoclastogenic cytokines” [50].

Other studies have shown that moderate-intensity exercise performed on a regular basis decreases blood levels of osteoclastogenic cytokines and increases blood levels of antiosteoclastogenic cytokines. Santos et al. reported that exercise training of 22 elderly men for 60 min/day, 3 days per week for 24 weeks reduced blood levels of IL-6 and TNF-α and increased blood levels of IL-10 [51]. In a similar study involving 6 months of aerobic or resistance exercise in 80 elderly men, El-Kader et al. reported that aerobic exercise was superior to resistance exercise in reducing blood levels of IL-6 and TNF-α and increasing blood levels of IL-10 [52,53]. Similar results were reported by Yuan and associates, including exercise-related reductions in osteoclastogenic cytokines IL-1, IL-6, and TNF-α and exercise-related increases in antiosteoclastogenic cytokines IL-2, IL-10, IL-12, IL-13, IL-18, and IFN-γ [54]. In a study involving the effect of acute resistance knee exercise (25 sets of 10 repetitions at 60% of one repetition maximum) in 12 women with knee OA, Helmark et al. found that exercise increased intraarticular and perisynovial levels of the anti-inflammatory cytokine IL-10 as compared to levels found in 13 non-exercising controls [55].

In summary, exercise upregulates PBMC production and serum levels of antiosteoclastogenic cytokines and downregulates PBMC production and serum levels of osteoclastogenic cytokines. In one study, exercise increased intra-articular and perisynovial levels of IL-10 in patients with knee OA.

## 9. Scaffolds

A variety of scaffolds are in development for use in MACI therapy, including hydrogels containing type I collagen, hyaluronic acid, albumin, fibrin, agarose, or alginate, and composite scaffolds composed of type I and type III collagen, hyaluronic acid and poly-glycolic acid, polyacetic acid, or polydioxanone. Currently, clinical trial entry for osteochondral repair is only approved for a nanocomposite three-layered collagen-hydroxyapatite scaffold, a poly (lactic-co-glycolic acid)-calcium-sulfate bilayer scaffold, and an aragonite-based scaffold. There is emerging interest in scaffolds that can deliver genes or gene carriers that upregulate the production of growth factors in articular cartilage and subchondral bone [56].

## 10. Rehabilitation Protocols

### 10.1. For Osteoarthritis

There is no consensus on what constitutes an optimal rehabilitation exercise program for patients with OA with the exception that regularly performed physical exercise should become a lifetime commitment and should be done in sufficient volume to relieve pain and improve function. In designing a rehabilitation program consideration should be given to exercises that simulate the type of muscle activity that patients use in their daily routines. The American College of Sports Medicine (ACSM) guidelines suggest that aerobic training should include a minimum of 150 min of moderate intensity or 75 min of vigorous intensity aerobic exercise per week in bouts of at least 10 min. For resistance training, two sessions per week, with two sets of 8 to 12 repetitions at a load of 60% to 70% of one repetition maximum with a rest period of ≥ 48 h between resistance training sessions are indicated. Resistance training can produce favorable responses independent of the type of equipment (dynamometers, weights, bands) utilized, the type of exercise (e.g., isokinetic, isotonic), and the muscle action (i.e., isometric, eccentric concentric) performed [57].

### 10.2. For Post-Transplant Rehabilitation

In their comprehensive review of postoperative procedures used to rehabilitate matrix-induced autologous chondrocyte implants of the tibiofemoral joint, Edward and associates concluded that allowing patients to bear full weight at 6 weeks post-operation allowed a quicker return to normal activity. Their review included 6-, 8-, 10-, and 12-week rehabilitation programs, all of which began with two weeks of 20% weight bearing coupled with continuous passive motion (CPM) of 0–40 degree knee flexion exercises starting 12–24 h post-surgery [58]. Upon full recovery from transplant surgery, applying the ACSM exercise guidelines for rehabilitation of osteoarthritis would seem appropriate.

## 11. Pre-Implantation Exercises

For healthy donors of bone marrow-derived MSCs the author recommends prescribing an aerobic exercise regimen using the following guidelines: 1. Calculate the patient’s age-based maximum heart rate (MHR) using the following formula: MHR = 220 − age. 2. Start the exercise program at 40–50% of MHR with weekly increments until the patient reaches 75–85% of MHR. The exercises should be done ≥ 30 min/day, 5 days/week. This exercise formula may also be useful in setting aerobic exercise goals in OA patients and in patients with implants following their period of rehabilitation.

## 12. Discussion

Mesenchymal stem cells are fibroblast-like cells that arise from embryonic mesenchyme and serve as the source of bone, cartilage, tendon, and adipose tissue. In addition to bone marrow and peripheral blood, MSCs are found in the vascular niches of adipose tissue, skeletal and cardiac muscle, lung, cartilage, and tendons. These progenitor cells synthesize and secrete a number of growth factors, extracellular matrix proteins, and cytokines that support the growth and survival of hematopoietic stem cells. They are identified by their expression of cell surface markers CD73, CD90, and CD105 and the absence of hematopoietic markers CD14 and CD45. Other defining criteria for MSCs include evidence of clonal expansion and/or the capacity to differentiate into multiple cell types, including tendon, cartilage, bone, and adipose tissue. MSCs represent a small fraction of the mononuclear cell population in bone marrow (0.001–0.01%) [59].

At present, implant techniques used to treat knee OA include third-generation scaffold implantations and chondrocyte and MSC injectable techniques. Importantly, methods using biomaterials that incorporate chondroprogenitors, chondrocytes, and MSCs have led to significant progress in regenerating OA joints. Scaffold implantation has produced a hyaline-like cartilage repair, durable pain and function improvement, and no donor site morbidity. BM-MSC injections have resulted in pain and functional improvement but have limited evidence of efficacy [11].

There is an emerging interest in developing scaffolds using viral and nonviral vectors that deliver genes or gene products that stimulate the production of growth factors by articular chondrocytes and subchondral bone. These growth factors include TGF-β, IGF-1, BMPs (particularly BMP-2 and BMP-7), and FGF-2. The use of transcription factors to promote osteochondral healing, including *Sox5, Sox6, Sox9*, *Runx2*, and Osx is planned, as well as the use of mRNAs [56]. It is reassuring that physical exercise and strain studies have been shown to achieve similar goals, including the upregulation of the expression of osteochondral genes and transcription factors (*Runx2,* Osx, *MSx1, SPP-1, Sox9, COL2A1, ATG3, and Ulk1),* mRNAs encoding ALP, BMP2, BMP4, OCL, and collagen I, and the production of osteochondral end products including TERT, ALP, caspase3, osteocalcin, BMP2, and BMP6 (see Figure 2, Figure 3 and Figure 4).

In light of the beneficial effects of exercise on articular cartilage chondrocytes and on the BM-MSC expression of osteochondral genes, transcription factors, and products, it is anticipated that exercise will improve the results of ACIs and MACIs in patients with osteochondral defects. ACIs involve an arthroscopic biopsy of a small piece of articular cartilage taken from an area not subjected to major pressure, culturing the chondrocytes and injecting them into the diseased joint of the donor/recipient [56]; in MACIs, the chondrocytes are incorporated into a scaffold matrix and placed in the osteochondral defect during an open procedure. Preclinical trials have shown that both ACIs and MACIs are successful in producing hyaline-like cartilage regrowth [60,61,62,63] with reasonable long-term durability [64]. However, ACIs are associated with up to 40% dedifferentiation of chondrocytes during culturing and/or after the transplant [65]. Whether exercise will improve this aspect of ACIs is yet to be determined.

Whether other joint preservation techniques are benefitted by exercise is also to be determined. Osteoplasty involves the drilling or punching of holes through the subchondral plate at the site of the chondral defect; this incites an inflammatory response which includes the mobilization of BM-MSCs to the articular surface [66]. Since exercise has been shown to increase circulating levels of BM-MSCs, exercise done before and after osteoplasty has the potential to improve the results of this regenerative technique.

The evidence provided in this review supports a policy of recommending physical exercise for autologous chondrocyte donors/recipients and BM-MSC donors and recipients. In the author’s view, patients with OA and recipients of osteochondral implants should make a lifetime commitment to perform physical exercise in keeping with ACSM guidelines.

Recommending exercise for patients undergoing regenerative procedures has the added benefit of reducing their risks for ischemic cardiovascular disease, hypertension, diabetes mellitus, metabolic syndrome, and certain forms of cancer [67].

## 13. Conclusions

Physical exercise done both by bone marrow-derived mesenchymal stem cell donors and recipients and by autologous chondrocyte donor/recipients may improve the outcome of osteochondral regeneration therapies and improve skeletal health by downregulating the production of osteoclastogenic cytokines and upregulating the production of antiosteoclastogenic cytokines.
